# Preliminary Research towards Acute Effects of Different Doses of Caffeine on Strength–Power Performance in Highly Trained Judo Athletes

**DOI:** 10.3390/ijerph19052868

**Published:** 2022-03-01

**Authors:** Robert Krawczyk, Michal Krzysztofik, Maciej Kostrzewa, Zuzanna Komarek, Michal Wilk, Juan Del Coso, Aleksandra Filip-Stachnik

**Affiliations:** 1 Institute of Sport Sciences, The Jerzy Kukuczka Academy of Physical Education in Katowice, 40-065 Katowice, Poland; r.krawczyk@awf.katowice.pl (R.K.); m.krzysztofik@awf.katowice.pl (M.K.); m.kostrzewa@awf.katowice.pl (M.K.); m.wilk@awf.katowice.pl (M.W.); 2 Nutrition and Sports Performance Research Group, The Jerzy Kukuczka Academy of Physical Education in Katowice, 40-065 Katowice, Poland; 43680@awfkatowice.edu.pl; 3 Centre for Sports Studies, Rey Juan Carlos University, 28942 Fuenlabrada, Spain

**Keywords:** performance-enhancing substances, martial arts, athletes, athletic performance, caffeine

## Abstract

Although several previous studies examined the effect of pre-exercise caffeine ingestion on judo-specific performance, the optimal dose of caffeine to maximise the ergogenic effects for judoka is not clear. The main purpose of this study was to analyse the effects of oral administration of 3 and 6 mg/kg of caffeine on a battery of physical tests associated with judo performance. Ten highly trained national-level judoka (6 men and 4 women, age: 24.1 ± 4.7 years, body mass: 73.4 ± 12.9 kg, 15.1 ± 5.2 years of judo training experience, 2.6 mg/kg/day of habitual caffeine intake) participated in a randomized, crossover, placebo-controlled and double-blind experiment. Each judoka performed three identical experimental sessions after: (a) ingestion of 3 mg/kg of caffeine (CAF-3); (b) ingestion of 6 mg/kg of caffeine (CAF-6); (c) ingestion of a placebo (PLAC). After 60 min for substance absorption, participants performed the following tests: (a) bench press exercise with 50% of the load representing one-repetition maximum (1RM), including three sets of three repetitions; (b) bench pull exercise with 50% of 1RM including three sets of three repetitions; (c) countermovement jump; (d) maximal isometric handgrip strength test; (e) dynamic and isometric versions of the Judogi Grip Strength Test. In comparison with PLAC, the ingestion of CAF-3 and CAF-6 increased peak bar velocity in the bench press exercise (1.27 ± 0.11 vs. 1.34 ± 0.13 and 1.34 ± 0.15 m/s, respectively; *p* < 0.01) and mean bar velocity in the bench pull exercise (1.03 ± 0.15 vs. 1.13 ± 0.13 and 1.17 ± 0.15 m/s; *p* < 0.05). Only CAF-6 increased mean bar velocity in the bench press exercise when compared with PLAC (0.96 ± 0.09 vs. 1.02 ± 0.11 m/s; *p* < 0.05). Both CAF-3 and CAF-6 significantly increased the number of repetitions in the Judogi Grip Strength Test (17 ± 10 vs. 20 ± 10 and 20 ± 10 repetitions; *p* < 0.05). There were no differences between PLAC and caffeine doses in the remaining tests. The pre-exercise ingestion of 3 and 6 mg/kg of caffeine effectively obtained meaningful improvements in several aspects associated with judo performance. From a practical viewpoint, the selection between 3 or 6 mg/kg of caffeine may depend on previously tested individual responses during simulated competition.

## 1. Introduction

Judo is a grappling sport characterised by intermittent bursts of high-intensity muscular activity [1], and it has been an Olympic discipline since 1964. Depending on the sports level, judo contests have different rules, including the duration of contests and rounds. However, the ultimate goal is for a judoka (i.e., the athletes practising judo) to score an ippon whereby they are awarded victory immediately, and which may be achieved via throwing, immobilisation, strangle, or elbow-lock techniques [2]. From a physical point of view, a judoka needs to develop various physical abilities to excel in this sport, including anaerobic and aerobic capacity, muscle power, strength–endurance, and maximal isometric and dynamic strength [1]. To apply judo techniques or control the opponent, it is key for one to obtain a grip on the opponent’s *judogi* (while avoiding a rival’s attempt to grip one’s own *judogi*). For this reason, almost half of the fighting time is spent in gripping disputes, which results in high levels of fatigue in the upper limbs. Hence, both the development of maximal handgrip strength and strength–endurance in the upper limbs are very relevant factors for judo success [3,4]. Once the grip is obtained, the success of judo throwing techniques is primarily associated with fast movements and power production in the lower limbs. Establishing superiority in grip disputes allows judoka to control their opponents sufficiently to execute throwing techniques, which are dependent on power production in the lower limbs [5]. Thus, high muscle performance qualities (i.e., maximal muscle strength, muscle endurance, and power) of upper and lower limbs are key performance indicators for judo success [1].

Given the physiological demands of judo, it is likely caffeine could be an effective ergogenic aid [6]. It is well established that acute caffeine intake may enhance muscular endurance [7], maximal strength [8,9], muscle power [8,10,11], and isometric strength [7] in a wide range of athletes. Additionally, several studies have examined the effects of acute caffeine intake on judoka [12,13,14,15,16,17,18,19,20,21]. Still, the results are conflicting due to the differences in caffeine doses and testing used to assess judo performance. In several investigations, caffeine supplementation between 4 and 9 mg of caffeine per kg of body mass (i.e., mg/kg) significantly improved performance during the Special Judo Fitness Test [12,14] and during simulated judo matches [14], increased dynamic strength during a gripping judogi test [17], enhanced maximal isometric handgrip strength test [17], and improved running during a 5 m multiple shuttles running test [20]. On the other hand, some studies did not find a positive effect of caffeine intake (between 3 and 6 mg/kg) on the Special Judo Fitness Test [14,15,16], on variables associated to combat activity [19], on cycling power during the Wingate test [21], or on the power output of upper and lower libs and isometric strength [22]. This variation may arise from the administration of different caffeine doses, the use of different physical tests, and the recruitment of study participants with varying performance levels (from junior- to international-level judoka; graduation range between brown and black). 

However, the analysis of these previous investigations does not clearly reveal why some investigations presented performance benefits of acute caffeine intake while others did not reveal such benefits. This is because different dose of caffeine and forms of administration improved performance in judo-specific tests and in participants with varying performance levels, while the same doses of caffeine were not effective in other investigations with similar testing and participants. Of note, only two studies [14,23] used multiple doses of caffeine (3, 6, and 9 mg/kg of caffeine from pills and ~2.7 and ~5.4 mg/kg of caffeine from chewing gum) in highly trained judoka. In those investigations [14,23], there was a tendency for a dose-specific improvement in the number of throws performed during the Special Judo Fitness Test [14,23] and in the number of offensive actions during a simulated match [14] along with the dose. It is important to note that not all individuals obtained benefits from acute caffeine intake of similar magnitude. However, the individual variations in response to caffeine intake have not been previously studied in judo. Interestingly, two previous investigations [14,23] indicated that habitual caffeine intake of judoka might reduce responses to acute caffeine ingestion. For these reasons, testing the potential ergogenic benefits of various doses of caffeine on judo performance may contribute to understand the optimal dose of caffeine and to create practical recommendations for judoka.

Given the highly individual nature of athletic responses to caffeine, the aim of this investigation was to analyse the effects of oral administration of 3 and 6 mg/kg of caffeine on a battery of physical tests associated with judo performance. Taking into account that all scoring actions in judo depend on the grip in the opponent’s *judogi* [3], we analysed maximal isometric handgrip strength and performance during a Judogi Grip Strength Test. As the upper limbs are constantly performing pulling and pushing movements to set up attacks [24] and judo throwing techniques are dependent on the power output of lower limbs [3,5], we also assessed power output during the bench press, bench pull exercise, and in a countermovement jump. Considering that the ergogenic benefits of caffeine might depend on the training status [25] and very few studies have been undertaken on highly trained athletes [26], we included only elite judoka as the study participants. We hypothesised that the ingestion of the two caffeine doses would enhance performance in all the applied tests in a dose-dependent manner.

## 2. Materials and Methods

### 2.1. Study Athletes

Ten healthy and experienced judoka (6 men and 4 women) participated in the study. Judoka were categorized as highly trained national-level because they occupied leading positions in the Polish national ranking. All of them were members of the Polish national team. Main characteristics of the study sample are depicted in Table 1. Study was conducted during the competitive season to ensure that judoka were acting near their peak physical fitness. The inclusion criteria were as follows: (a) free from neuromuscular and musculoskeletal disorders; (b) black belt in judo; (c) not ingesting any medications, dietary supplements, or ergogenic aids which could potentially affect the study outcomes; (d) self-described satisfactory health status. Judoka were excluded if they reported (a) positive smoking status; or (b) possible allergy to caffeine. All participants had prior judo experience of at least ten years and had trained a minimum of 2 h/day, 6 days/week during the previous year (competitive season). Participants were classified as mild caffeine users according to the previously proposed classification [27]. The study protocol was approved by the Academy Ethics Committee per the latest version of the Declaration of Helsinki. All judoka provided their written informed consent prior to participation in this study.

### 2.2. Pre-Experimental Standardization

Before the first testing session, judoka were instructed to maintain their dietary patterns (including a pre-workout meal) and their habitual caffeine intake during the study period. The diet standardization protocol was the same as in a previous study examining the impact of two caffeine doses on exercise performance [28]. Judoka were also asked to avoid caffeine, alcohol, and strenuous exercise 24 h before each data collection.

### 2.3. Experimental Design

**Figure 1:**
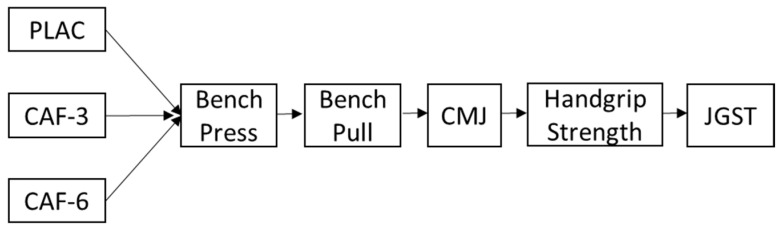
Study design. PLAC: placebo; CAF 3: a dose of 3 mg/kg of caffeine; CAF-6: a dose of 6 mg/kg of caffeine; CMJ: countermovement jump; JGST: Judogi Grip Strength Test.

To explore the effect of different caffeine doses on a battery of physical tests associated with judo performance, the judoka participated in a randomized, double-blind, placebo-controlled crossover experiment where each judoka acted as his/her own control. Each judoka participated in one familiarization session and then in three identical experimental trials after ingestion: (a) placebo (PLAC); (b) 3 mg/kg of caffeine (CAF-3); (c) 6 mg/kg of caffeine (CAF-6). The trials were separated by a minimum of 72 h to allow complete recovery and substance wash-out. During each trial, judoka performed an identical self-selected warm-up and then performed the following physical tests in this order: (a) bench press exercise performed at maximal velocity with 50% of one-repetition maximum (1RM), including 3 sets of 3 repetitions); (b) bench pull exercise at maximal velocity with 50% of 1RM, including 3 sets of 3 repetitions); (c) a countermovement jump; (d) maximal isometric handgrip strength; (e) dynamic and isometric versions of the Judogi Grip Strength Test (Figure 1). Once the testing was finished, judoka were asked about the presence of side effects habitually associated with caffeine intake during the trial and 24 h after the testing. The effectiveness of blinding was checked post-exercise. All testing was conducted at the Strength and Power Laboratory of the Academy of Physical Education in Katowice, Poland, under controlled ambient conditions (~21 °C).

### 2.4. Familiarization Session and One Repetition Maximum Test

During the familiarization session, judoka visited the laboratory at the same time of day as in the following testing sessions. On this day, bioimpedance analysis (model 370, InBody, Biospace Co., Seoul, Korea) was used to evaluate body composition. Then, judoka performed a fifteen-minute warm-up followed by a 1RM test in barbell bench press and barbell bench pull exercise [29] according to previous guidelines [30,31]. The 1RM value was achieved within 5 trials to be considered valid. Bench press and bench pull exercises were performed with a grip width according to previous guidelines and controlled during the study period [32]. The 1 RM tests were executed with an in-between 30 min rest interval. At the end of this testing, participants were familiarized with all performance tests to be carried out in the experimental trials.

### 2.5. Battery of Performance Tests

#### 2.5.1. Movement Velocity in the Barbell Bench Press and Barbell Bench Pull Exercise

After the warm-up, which was the same as in the familiarization trial, judoka performed 3 sets of 3 repetitions at 50% of their 1RM in the bench press exercise. We selected this type of testing with several sets and repetitions per sets to replicate some of the conditions used during strength training [33] and to understand the potential effects of caffeine during strength training in judoka. All repetitions during bench press were performed without intentionally pausing between the positive and negative phase of exercise, without raising the hips off the bench, and without bouncing the barbell off the chest. The sets were performed with a 3 min rest interval. Three min after finishing the bench press exercise, judoka performed 3 sets of 3 repetitions at 50% 1RM in the bench pull exercise. In the bench pull exercise, judoka were instructed to lie prone and place their chin on the padded edge of a high bench. The pulling phase began with both elbows in full extension. The judoka were instructed to pull with maximum effort until the barbell struck the underside of the bench, after which it was again lowered to the starting position. Participants were not allowed to use their legs to hold onto the bench. A momentary pause, which lasted approximately 1.5 s, was interposed between the positive and negative phases of bench pull exercise to minimize the contribution of the rebound effect and allow for more reproducible, consistent measurements. Judoka were required to always perform the concentric and eccentric phase of both exercises explosively, at maximal voluntary velocity. A rotatory encoder (Tendo Power Analyzer, Tendo Sport Machines, Trencin, Slovakia) was used to instantly record bar velocity during the whole range of motion, as in previous investigations [34]. This device emerged as a reliable system for measuring bar velocity (between-session intra-class correlation coefficient (ICC) and coefficient of variation (CV); expressed as a percentage of the participants’ mean scores: of 0.97% and 9.6%, respectively, for mean velocity and 0.98% and 9.0% for peak velocity) [35]. Only the exercises’ concentric phase (pushing for the bench press and pulling for the bench pull) was analysed in the present study. During each repetition, peak bar velocity (peak velocity, in m/s) and mean bar velocity (mean velocity, in m/s) were registered and included into the analysis. Mean velocity was obtained as the mean of the three repetitions, while peak velocity was obtained from the best repetition for each 3-repetition set. In the current study, the within-session CV values (expressed as a percentage of the participants’ mean scores) for mean and peak bar velocity were 3.5–4.0% and 3.2–3.5%, respectively, during the bench press exercise and 3.9–6.9% and 4.6–8.3% during bench pull exercise, respectively. Within-sessions ICC obtained from best attempt of each set showed good reliability values for mean (ICC = 0.82–0.85) and peak bar velocity (ICC = 0.84–0.89) during bench press and moderate to good reliability values for mean (ICC = 0.71–0.86) and peak (ICC = 0.62–0.74) bar velocity during bench pull exercise.

#### 2.5.2. Jumping Performance Assessment

Following the bench pull, the judoka were provided with another three-minute rest interval. Then, the judoka performed a standardized additional lower-body warm-up that consisted of one minute of light running and ten bodyweight squats. Afterwards, judoka performed three countermovement jumps (CMJ) without arm swing [36]. The judoka reset to the starting position after each jump, and the procedure was completed for a total of 3 jumps. The best jump of the three attempts was used for the analysis. If judoka exhibited excessive knee flexion in the air, the jump was cancelled and repeated. Based on the measurements of flight time jump height (cm) was calculated. This test was performed on a force platform (Force Decks, Vald Performance, Australia), and it has been previously proposed as a reliable method for measuring jump height (between-sessions ICC = 0.93 and CV = 5.1%) [36]. In the current study, the within-session CV (expressed as a percentage of the participants’ mean scores) for jump high measures was 3.4–6.0%. Within-session ICC obtained from the best attempt of each set showed excellent reliability values for jump height (ICC = 0.95–0.98).

#### 2.5.3. Maximal Isometric Handgrip Strength

Following the CMJ, the judoka were provided with another three-minute rest interval. A Bremshey Sport^®^ electronic hand dynamometer (Almere, The Netherlands) was used to measure the maximum isometric handgrip strength of the dominant and non-dominant hand, as described elsewhere [37]. The highest value of the three repetitions was used for further analysis. The parameter used for analysis was peak strength (kg) of the dominant and non-dominant hands. Hand dominancy was determined by asking the judoka which hand does he/she normally use when gripping the opponent’s lapel (*tsurite*), while the non-dominant hand was assumed as controlling the opponent’s sleeve (*hikite*) [38]. In the current study, the within-session CV values for isometric handgrip strength in the dominant and non-dominant hands were 3.9–5.1% and 3.2–4.8%, respectively. Within-session ICC showed excellent reliability values for dominant (ICC = 0.94–0.97) and non-dominant hand (ICC = 0.94–0.97).

#### 2.5.4. Judogi Grip Strength Test

Following the handgrip strength test, judoka were provided with another three-minute rest interval. Then, athletes performed both the dynamic and isometric versions of the Judogi Grip Strength Test (JGST) according to a previous protocol [24]. After a 5 min interval, the judoka performed the isometric JGST, which consisted of sustaining a full elbow flexion for the maximal possible time. For this test, the judoka griped the *judogi* and rolled around a hanging bar as previously described, with the elbow joint at maximal flexion. The chronometer was stopped when the judoka could no longer maintain the original position. The total number of repetitions and time were considered as performance measurements from the dynamic and isometric tests, respectively. The between-sessions reliability of both JGSTs has been assessed in a previous study, presenting an ICC higher than 0.98 [39].

### 2.6. Side Effects

Immediately after each data collection and after 24 h, the judoka were asked about caffeine-associated side effects using a questionnaire with a yes/no response. Additionally, participants were asked about increased vigour/activeness perception of performance improvement during the testing [9,40,41].

### 2.7. Assessment of Blinding

The blinding efficiency was assessed post-exercise by asking the judoka the following question: “Which supplement do you think you have ingested”? This question had three possible responses: (a) “caffeine”, (b) “placebo”, and (c) “I do not know”.

### 2.8. Statistical Analysis

#### 2.8.1. Performance Tests

All calculations were performed using Statistica 13.3 (TIBCO Software Inc., Palo Alto, CA, USA) and are expressed as means with standard deviations (±SD). All variables presented a normal distribution according to the Shapiro–Wilk test. Statistical significance was set at *p* < 0.05. Verification of differences in calorie intake and macronutrients ingestion between conditions (i.e., PLAC vs. CAF-3 vs. CAF-6) was performed using one-way analysis of variance (ANOVA) for repeated measures. A one-way ANOVA of repeated measures was also used to analyse differences between conditions during maximal isometric handgrip strength, Judogi Grip Strength Test, and CMJ. Verification of differences between conditions in peak bar velocity (peak velocity) and mean bar velocity (mean velocity) during bench press and bench pull exercises was performed using a two-way ANOVA (condition × set) with repeated measures. We selected the two-way ANOVA because this statistical approach offers the possibility of measuring a main effect of condition (i.e., caffeine dosage vs. placebo), a main effect of set (i.e., changes across the three sets), or a condition × set interaction (i.e., the effect of caffeine changed across the three sets). In the case of a significant main effect, post hoc comparisons were conducted using Tukey’s tests to adjust for multiple comparisons. Percentage changes and 95% confidence intervals were also calculated. Effect sizes (ES) were calculated by using Cohen’s *d* [42]. The reliability of the battery of performance tests was assessed via within-session ICC analysis (independently for each session) using a single measure, two-way mixed, absolute-agreement parameters. Values less than 0.5, between 0.5 and 0.75, between 0.75 and 0.9, and greater than 0.90 indicate poor, moderate, good, and excellent reliability, respectively [43]. The CV (expressed as a percentage of the participants’ mean value) was calculated for intersession reliability. ICC and CV were calculated by using the best values obtained per each set.

#### 2.8.2. Side Effects

A two-way contingency table analysis was conducted to evaluate whether CAF dose was associated with the occurrence of side effects immediately and 24 h post-experiment (increased urine output, tachycardia and heart palpitations, anxiety or nervousness, headache, gastrointestinal problems, insomnia, increased vigour activeness, perception of performance improvement). The two variables were CAF dose with three possibilities (PLAC, CAF-3, CAF-6) and occurrence of the side effect with two possibilities (yes and no). Moreover, a Cochran’s Q test with pairwise comparison was calculated to examine the differences between doses in the occurrence of side effects.

#### 2.8.3. Assessment of Blinding

The effectiveness of the blinding was examined using the Bang’s Blinding Index (BBI). All analyses were performed using the R Statistical Software V 3.6.3 (RStudio Team, 2019) using the add-on package ‘BI’.

## 3. Results

### 3.1. Standardizations

The one-way ANOVA indicated no significant differences in energy intake (2852 ± 657, 2845 ± 598, 2795 ± 656 kcal/day; *p* = 0.65) and in the proportions of protein (1.6, 1.7, 1.6 g/kg; *p* = 0.532), carbohydrate (5.5, 5.5, 5.4 g/kg; *p* = 0.958), and fat (1.3, 1.2, 1.2 g/kg; *p* = 0.694) in the diet of the judoka for the 24 h before PLAC, CAF-3, and CAF-6 conditions, respectively.

### 3.2. Battery of Performance Tests

#### 3.2.1. Movement Velocity in the Bench Press and Bench Pull Exercise

The two-way ANOVA indicated a significant main effect of condition for mean (F = 3.910; *p* = 0.039) and peak bar velocity during the bench press exercise (F = 8.664; *p* = 0.002). The pairwise comparisons revealed a significant improvement in mean velocity for CAF-6 when compared with PLAC (1.02 ± 0.11 vs. 0.96 ± 0.09 m/s; *p* = 0.041). In peak velocity, the positive effect of caffeine over PLAC was identified for CAF-3 (1.27 ± 0.11 vs. 1.34 ± 0.13 m/s; *p* = 0.005) and for CAF-6 (1.34 ± 0.15 m/s; *p* = 0.006; Table 2). The results of the two-way repeated measures ANOVA indicated a significant effect of the set for peak velocity during a bench press exercise (F = 6.193; *p* = 0.009). The pairwise comparisons revealed a significant difference in peak velocity between the first and the second set (*p* = 0.005) and between the first and third set (*p* = 0.006) during bench press exercise. The results of the two-way repeated measures ANOVA indicated no significant effect of set (F = 3.017; *p* = 0.074) for mean velocity or set × condition interaction for mean (F = 0.735; *p* = 0.737) and peak (F = 2.126; *p* = 0.097) velocity during the bench press exercise.

The two-way ANOVA indicated a significant main effect of condition for mean velocity during a bench pull exercise (F = 8.088; *p* = 0.003), without differences in peak velocity (F = 2.185, *p* = 0.141). The pairwise comparisons revealed a significant improvement in mean velocity for CAF-3 over PLAC (1.13 ± 0.13 vs. 1.03 ± 0.15 m/s; *p* = 0.035) which was also present with CAF-6 (1.17 ± 0.15 m/s; *p* = 0.003; Table 2). The results of the two-way repeated measures ANOVA indicated no significant effect of set or set × condition interaction for mean (F = 3.230; *p* = 0.063; F = 0.744; *p* = 0.567) and peak (F = 0.782; *p* = 0.472; F = 0.461; *p* = 0.763) bar velocity during bench pull exercise.

#### 3.2.2. Jumping Performance Assessment

The one-way ANOVA did not reveal a significant main effect for condition on jump height (F = 0.588; *p* = 0.565; Table 3).

#### 3.2.3. Maximal Isometric Handgrip Strength

The one-way ANOVA did not reveal a significant main effect of condition for handgrip strength in the dominant (F = 1.637; *p* = 0.222) and non-dominant hand (F = 1.718; *p* = 0.207; Table 3). There was no effect of any caffeine condition when the sum of both sides was considered (F = 1.687; *p* = 0.213).

#### 3.2.4. Judogi Grip Strength Test

The one-way ANOVA found a significant main effect of condition for the number of repetitions during the dynamic Judogi Grip Strength Test (F = 9.259; *p* = 0.002; Table 3). The pairwise comparisons revealed a significant improvement in the dynamic Judogi Grip Strength Test for both CAF-6 (*p* = 0.003) and CAF-6 (*p* = 0.008) over PLAC, without any difference between CAF-3 and CAF-6 (*p* = 0.847). However, the one-way ANOVA did not find a significant main effect of condition for the gripping duration during the isometric Judogi Grip Strength Test (F = 0.131; *p* = 0.877).

### 3.3. Side Effects

A Fisher’s Exact Test showed no statistically significant associations between CAF dose and reported side effects immediately post-testing. For 24 h after testing, judoka did not report any side effects. Cochran’s Q test determined a statistically significant difference in the proportion of participants who reported increased vigour activeness immediately post-testing (*p* = 0.050), with no other statistically significant differences for the rest of the analysed side effects. Pairwise comparison showed that significantly more participants indicated increased vigour activeness post-testing with CAF-6 than PLAC (*p* = 0.043) but not between CAF-3 vs. CAF-6 and CAF-3 vs. PLAC (*p* = 0.662 for both).

### 3.4. Assessment of Blinding

From the total, 70% of judoka correctly identified the PLAC condition. In the CAF-3 and CAF-6 conditions, 40% and 50% of the judoka correctly identified caffeine administration, respectively. As determined by the BBI, the blinding of the participants was successful for all caffeine conditions, while during the PLAC condition judoka correctly guessed their allocation (Table 4).

## 4. Discussion

The present study aimed to compare the acute effects of soral ingestion of 3 and 6 mg/kg of caffeine on strength–power performance in highly trained national-level judoka. The results of the present study indicate that the ingestion of both 3 and 6 mg/kg of caffeine significantly increased peak bar velocity in the bench press exercise and mean bar velocity in the bench pull exercise, while 6 mg/kg of caffeine increased mean velocity in the bench press exercise. In addition, both doses of caffeine enhanced the number of repetitions performed during the dynamic Judogi Grip Strength Test. The results of the present study did not show any other significant ergogenic effects of the two caffeine conditions in the remaining tests. These outcomes suggest that ingestion of 3 and 6 mg of caffeine per kg of body mass was effective in obtaining meaningful improvements in several strength–power performance variables considered as determinant for judo performance. However, a clear dose response was not observed.

Several previous studies explored the effectiveness of acute caffeine administration on judo performance [12,13,14,15,16,18,19,21,23,44]. However, only three investigations [17,22,44] used performance tests to assess maximal grip strength and endurance. Gripping capacity in terms of strength and duration might be particularly important for judoka, as for gripping is the longest action performed during a match [45,46]. The results of the presented research showed that CAF-3 and CAF-6 improved the number of repetitions during the dynamic Judogi Grip Strength Test, which is in line with the results of a study by Lopes-Silva et al. [17]. Nevertheless, in the presented study, we did not observe a significant effect of caffeine in the isometric maximal handgrip strength over PLAC, which is contrary to findings of Diaz-Lara et al. [44]. In fact, in Diaz-Lara’s investigation [44], 3 mg/kg of caffeine improved the handgrip strength of the dominant and non-dominant hand, an effect that was not present in the current investigation with any of the caffeine doses. In the light of the current results, the benefits of caffeine-induced changes in gripping performance should be tested in more ecologically valid scenarios for combat sports in general and for judo in particular (e.g., between following fights during competition).

The present investigation included the measurement of bar velocity in the bench press and bench pull exercises (corresponding to explosive pushing–pulling actions during judo combat [24]) and assessment of power output of lower body (corresponding with effectiveness of throwing techniques [3,5]). Although numerous studies analysed the effect of caffeine on bar velocity during the bench press exercise [10,40,47,48,49,50], to the best of our knowledge, this is the first study which assessed such effects in a bench pull exercise. Results of the present study show that both doses of caffeine enhanced peak and mean bar velocity during the bench press and bench pull exercise, which is in line with results of several previous studies which analysed the movement velocity during resistance exercise [10,40,47,48,49,51]. Interestingly, we did not observe an improvement in jump height during the countermovement jump, which is contrary to previous findings [8,49,50]. However, the high training status of participants or the individual differences in the use of the stretch-shortening cycle during countermovement jump might be possible explanations of lack of changes.

The magnitude of the ergogenic effects of caffeine can also be related to caffeine dose. In the present study, we explored the acute effects of CAF-3 and CAF-6, considering that they are in the range of the most commonly used doses in sports performance research [52]. The results of our study failed to identify a clear dose–response, although some trends may be evident for the bench pull exercise and the maximal isometric handgrip strength test. However, the dose–response effect was not evident in other performance tests, suggesting that higher doses of caffeine may be effective only for some specific fitness performance test, although the most plausible explanation may be associated with the effect that other factors (i.e., doses in comparison to participant’s to level of habituation to caffeine, the population’s training status, etc.) might have on the obtained results Although several previous studies compared the effectiveness of different caffeine doses in jump height [50,53,54], upper body power output [40,50], and muscle endurance [9,55,56], the direct comparison with previous results is challenging, given the differences in the experimental protocols. Thus, from a practical standpoint, it seems that oral administration of both 3 and 6 mg/kg of caffeine was similarly effective when ingested before the physical performance in this population. Hence, the administration of the minimal effective dose (i.e., 3 mg/kg) may be advised when searching for increased performance with caffeine supplementation.

The habituation of participants to caffeine through chronic exposure to this substance might have influenced the ergogenic effect of acute caffeine intake [57]. Several previous studies explored the impact of habituation to caffeine on the effects of acute caffeine ingestion and showed conflicting results [49,50,55,58,59,60,61]. In some investigations, positive effects of caffeine have been observed in habituated participants [49,50,59,60], although several studies have reported diminished effects in such groups [9,40,55,58,61]. In fact, the results of those studies [49,50,59,60] lead to the conclusion that habitual caffeine intake does not impact the ergogenicity of caffeine, which is partially in line with the results of current investigations conducted on low caffeine users. However, it is still possible that the effect of this substance is higher in unhabituated individuals. Thus, the diminished effect of the impact of caffeine observed in several tests conducted in this investigation (i.e., bench pull exercise, jumping performance, handgrip test) in comparison with previous studies might be caused by habituation to caffeine. Moreover, it is probable that the sample size of the current investigation limited the obtained differences for various levels of habitual caffeine intake, and further studies should involve a larger sample size to confirm those findings.

The current study has some potential limitations: (1) the study did not include any biochemical analysis, such as plasma/urinary caffeine level, which could have aided in the explanation of the direct causes of performance changes; (2) the study was conducted on highly trained judoka, and generalizations of these results for judoka with another training background should be made with caution; (3) we assessed the impact of caffeine in mild habitual caffeine users, and future studies need to include judoka with different levels of habitual caffeine intake, particularly naïve caffeine users; (4) only within-session reliability values were obtained, and between day reliability coefficients are unknown for the tests used in this investigation. The study examined the effects of caffeine ingestion on a battery of physical tests associated with judo performance. However, judo is a complex sport comprising a number of specific skills which were not replicated in the tests used in this investigation. Future studies need to include protocols that mimic real-life judo competition to assure that the caffeine doses investigated here actually impact judo performance. Lastly, considering the deficiency of data on the effect of supplementation protocols in female athletes [62], future studies should assess and compare the effectiveness of different doses of caffeine in a homogenous sample of women judoka.

## 5. Conclusions

The results of the current investigation show that the oral administration of 3 and 6 mg/kg of caffeine significantly increased peak bar velocity in the bench press exercise, while only 6 mg/kg of caffeine increased mean velocity in the bench pull exercise. In addition, both 3 and 6 mg/kg of caffeine enhanced the number of repetitions performed during the dynamic Judogi Grip Strength Test. Results of the maximal handgrip strength of both the dominant and non-dominant hands, the grip duration during isometric Judogi Grip Strength Test, and countermovement jump height were similar under the two caffeine conditions with respect to placebo. In conclusion, the ingestion of 3 and 6 mg/kg of caffeine was effective for obtaining meaningful improvements in several aspects associated with judo performance. From a practical viewpoint, the benefits of pre-exercise ingestion of both caffeine doses seem to be similar, at least in highly trained judoka. In general, sports practitioners in the context of judo should consider the selection of the minimal effective dose of caffeine (i.e., 3 mg/kg) although the selection between 3 or 6 mg/kg of caffeine may depend on previously tested individual responses during simulated competition.

## Figures and Tables

**Table 1:** Main participants’ characteristics.

Variable (Units)	Men (*n* = 6)	Women (*n* = 4)
Age (years)	26.4 ± 5.3	20.8 ± 1.5
Body mass (kg)	76.0 ± 11.8	69.6 ± 15.6
Height (cm)	176.1 ± 4.1	167.0 ± 4.8
Body fat (%)	11.9 ± 5.3	20.1 ± 9.7
Judo experience (years)	17.5 ± 5.6	11.5 ± 3.1
Bench press exercise 1RM (kg)	110.8 ± 17.7	62.5 ± 11.9
Bench pull exercise 1RM (kg)	101.7 ± 8.8	65.0 ± 10.0
Habitual caffeine intake (mg/kg/day)	2.6 ± 2.2	2.7 ± 3.2
Energy intake (kcal/day)	3347 ± 230	2056 ± 202
Protein (g/kg/day)	1.9 ± 0.5	1.2 ± 0.4
Fat (g/kg/day)	1.4 ± 0.3	1.0 ± 0.3
Carbohydrates (g/kg/day)	6.3 ± 1.3	4.2 ± 0.7

Data reported as mean ± standard deviation.

**Table 2:** Peak and mean bar velocity for each set of the three sets of the bench press and bench pull exercises with the ingestion of 3 and 6 mg/kg of caffeine or with a placebo.

Conditions		Set 1	Set 2	Set 3
Mean Bar Velocity in the Bench Press Exercise (m/s)				
PLAC		0.94 ± 0.09	0.98 ± 0.09	0.97 ± 0.09
CAF-3		1.00 ± 0.11 *	1.01 ± 0.07	1.01 ± 0.08
CAF-6		1.00 ± 0.10 *	1.04 ±0.11 *	1.01 ± 0.11 *
ES (95% CI)	PLAC vs. CAF-3	0.60 (−0.32, 1.47)	0.37 (−0.53, 1.24)	0.47 (−1.34, 0.44)
	PLAC vs. CAF-6	0.63 (−1.5, 0.29)	0.60 (−1.47, 0.32)	0.40 (−1.27, 0.50)
Peak bar velocity in the Bench Press exercise (m/s)				
PLAC		1.26 ± 0.11	1.29 ± 0.11	1.25 ± 0.11
CAF-3		1.31 ± 0.15	1.35 ± 0.11	1.37 ± 0.12 *
CAF-6		1.32 ± 0.14 *	1.37 ± 0.14*	1.34 ± 0.16 *
ES (95% CI)	PLAC vs. CAF-3	0.38 (−1.25, 0.52)	0.55 (−1.41, 0.37)	1.04 (−1.93, -0.07)
	PLAC vs. CAF-6	0.56 (−1.42, 0.36)	0.95 (−1.83, 0.01)	0.66 (−1.53, 0.27)
Mean bar velocity in the Bench Pull exercise (m/s)				
PLAC		1.03 ± 0.16	1.00 ± 0.14	1.05 ± 0.15
CAF-3		1.11 ± 0.14	1.12 ± 0.12 *	1.15 ± 0.13 *
CAF-6		1.14 ± 0.15	1.19 ± 0.15 *	1.18 ± 0.15 *
ES (95% CI)	PLAC vs. CAF-3	0.53 (−0.38, 1.40)	0.92 (−0.04, 1.80)	0.71 (−1.58, 0.22)
	PLAC vs. CAF-6	0.71 (−1.58, 0.22)	1.31 (−0.30, 2.21)	0.87 (−1.74, 0.08)
Peak bar velocity in the Bench Pull exercise (m/s)				
PLAC		1.51 ± 0.24	1.53 ± 0.21	1.54 ± 0.19
CAF-3		1.51 ± 0.17	1.56 ± 0.16	1.58 ± 0.14
CAF-6		1.63 ± 0.18	1.59 ± 0.28	1.64 ± 0.21
ES (95% CI)	PLAC vs. CAF-3	0.00 (−0.88, 0.88)	0.16 (−1.03, 0.72)	0.24 (−1.11, 0.65)
	PLAC vs. CAF-6	0.57 (−1.43, 0.35)	0.24 (−1.11, 0.65)	0.50 (−1.37, 0.41)

All data are presented as mean ± standard deviation. PLAC: placebo; CAF-3: caffeine 3 mg/kg; CAF-6: caffeine 6 mg/kg. ES: effect size. CI: confidence interval; * statistically difference from placebo at *p* < 0.05.

**Table 3:** Results of the effects of caffeine on the exercise performance outcomes.

Variable	PLAC	CAF-3	CAF-6	PLAC vs. CAF-3		PLAC vs. CAF-6	
				ES (95% CI)	Relative Effect	ES (95% CI)	Relative Effect
Maximal isometric handgrip strength							
Peak absolute strength of dominant hand (kg)	46.2 ± 10.9	47.1 ± 10.2	48.4 ± 12.1	0.09 (−0.96, 0.80)	1.9%	0.19 (−1.06, 0.70)	4.7%
Peak absolute strength of non-dominant hand (kg)	45.6 ± 8.3	44.8 ± 8.7	46.7 ± 9.3	−0.09 (−0.97, 0.79)	−1.7%	0.12 (−0.76, 1)	2.4%
Peak absolute strength of both hands (kg)	91.8 ± 10.9	91.9 ± 10.2	95.10 ± 12.1	0.01 (−0.87, 0.89)	0.1%	0.29 (−0.61, 1.16)	3.6%
Countermovement jump							
Jump height (cm)	40.2 ± 11.6	41.1 ± 9.7	41.0 ± 10.3	0.08 (−0.80, 0.96)	2.3%	0.07 (−0.81, 0,95)	1.9%
Judogi Grip Strength Test							
Dynamic test (repetition)	17 ± 10	20 ± 10 *	20 ± 10 *	0.30 (−0.59, 1.17)	20.9%	0.30 (−0.59, 1.17)	18.0%
Isometric test (s)	47.0 ± 10.4	47.5 ± 11.2	47.9 ± 11.6	0.05 (−0.83, 0.92)	1.2%	0.08 (−0.80, 0.96)	1.9%

Data reported as mean ± standard deviation. ES: effect size; PLAC: placebo; CAF-3: 3 mg/kg of caffeine; CAF-6: 6 mg/kg of caffeine; * statistically significant difference from placebo at *p* < 0.05.

**Table 4:** Results of the assessment of blinding.

Condition	Responded as Placebo	Responded as Caffeine	Responded as Don’t Know	Bang’s Blinding Index Mean (95% CI)
PLAC	7	0	3	0.7 (0.46–0.94)
CAF-3	5	4	1	−0.1 (−0.59–0.39)
CAF-6	4	5	1	0.1 (−0.39–0.59)

PLAC: placebo; CAF-3: 3 mg/kg of caffeine; CAF-6: 6 mg/kg of caffeine. CI: confidence interval.

## Data Availability

The datasets used and/or analysed during the current study are available from the corresponding author on reasonable request.
